# Feasibility of increasing calcium content of drinking tap water following quality regulations to improve calcium intake at population level

**DOI:** 10.12688/gatesopenres.15184.2

**Published:** 2024-08-29

**Authors:** Natalia Matamoros, María Bernardita Puchulu, Jorge E Colman Lerner, Eduard Maury-Sintjago, Jorge L López, Verónica Sosio, José M Belizán, Andrés Porta, Gabriela Cormick

**Affiliations:** 1 Instituto de Desarrollo e Investigaciones Pediátricas "Prof. Dr. Fernando E. Viteri" Hospital de Niños Sor María Ludovica (IDIP), Instituto de Desarrollo e Investigaciones Pediátricas "Prof. Dr. Fernando E. Viteri" Hospital de Niños Sor María Ludovica (IDIP), Ministerio de Salud/Comisión de Investigaciones Científicas de La Provincia de Buenos Aires, La Plata, Buenos Aires Province, 1900, Argentina; 2 Universidad Nacional Arturo Jauretche (UNAJ), Florencio Varela, Buenos Aires, Argentina; 3 Facultad de Medicina, Departamento de Ciencias Fisiológicas, Universidad de Buenos Aires, Buenos Aires, 1121, Argentina; 4 Universidad Nacional de La Matanza, San Justo, Provincia de Buenos Aires, Argentina; 5 Centro de Investigación y Desarrollo en Ciencias Aplicadas "Dr. Jorge J. Ronco" (CINDECA), CONICET, La Plata, 1900, Argentina; 6 Facultad de Ciencias Exactas, Universidad Nacional de La Plata (UNLP), La Plata, Argentina; 7 Department of Nutrition and Public Health, Universidad del Bío-Bío, Chillan, 3800708, Chile; 8 Centro de Investigaciones en Epidemiología y Salud Pública (CIESP), CONICET, Ciudad de Buenos Aires, Argentina; 9 Departamento de Hidráulica, Facultad de Ingeniería, Universidad Nacional de La Plata (UNLP), La Plata, 1900, Argentina; 10 Instituto de Efectividad Clinica y Sanitaria, Buenos Aires, Argentina; 11 Centro de Investigaciones del Medio Ambiente (CIM), CONICET, La Plata, 1900, Argentina

**Keywords:** calcium; calcium chloride; drinking water; low-and middle-income country; water quality

## Abstract

**Background:**

Calcium intake is below recommendations in several parts of the world. Improving calcium intake has benefits not only for bone health but also helps to prevent pregnancy hypertension disorders. Calcium concentration of tap water is usually low The aim of the present study was to determine the maximum amount of calcium that can be added to tap water while complying with drinking water Argentine regulations.

**Methods:**

Tap water samples were collected from the Province of Buenos Aires (Argentina). Physicochemical properties and saturation index were measured. Different incremental concentrations of calcium chloride were added to the experimental aliquots.

**Results:**

Baseline water had a mean calcium concentration of 22.00 ± 2.54 mg/L, water hardness of 89.9 ± 6.4 mg/L CaCO _3_, and a saturation index of -1.50 ± 0.11. After the addition of 0.4554 ± 0.0071 g of salt, water hard-ness reached 355.0 ± 7.1 mg/L CaCO _3_, a calcium concentration of 140.50 ± 2.12 mg/L, and a saturation index -0.53 ± 0.02.

**Conclusions:**

This study shows that at laboratory level it is feasible to increase calcium concentration of drinking water by adding calcium chloride while complying with national standards. Calcium concentration of drinking tap water could be evaluated and minimum calcium concentration of tap water regulated so as to improve calcium intake in populations with low calcium intake.

## Introduction

Calcium is one of the inorganic elements in the human body involved in many vital functions, influencing many extracellular and intracellular processes ^1– 3^. Calcium is essential for development and growth. In addition, optimal calcium intake is necessary for bone health at all stages of life ^2, 4, 5^. Furthermore, appropriate calcium intake has shown many other health benefits such as those related to reduction of hypertensive disorders of pregnancy ^3, 6– 8^. Despite the benefits of calcium intake, intake values are well below recommendation in many parts of the world ^3, 9– 11^. Thus, it is imperative to consider other sources of calcium that may contribute to calcium recommendations.

Calcium intake is usually associated with dairy products intake; however, the impact that these foods have on total calcium intake depends on food consumption patterns ^12, 13^. Drinking water naturally contains calcium, however, the contribution of water minerals to total intake isseldom considering calcium bioavailability is comparable to calcium from dairy products ^2, 14, 15^.

Studies have reported that calcium-rich water significantly contributes to daily calcium intake in adults ^16– 20^. Calcium in water may be an efficient way to provide and improve calcium intake in countries that report low calcium intake ^15^. There are studies of the use of water as a fortification vehicle for different micronutrients such as fluoride ^21– 24^, iodine ^25^, iron ^26^, and ascorbic acid ^27^. The literature showed beneficial effects of calcium-rich water on biochemical parameters of bone metabolism ^28, 29^. Moreover, some studies have shown that cooking with high calcium water could avoid calcium loss from foods and increase calcium concentration of cooked foods ^30, 31^. A simulation analysis performed in 62 Low-and Middle-Income Countries (LMICs) revealed that if calcium concentration in tap and bottled water was increased at levels of 100 mg/L and 400 mg/L, respectively, an extra nine countries could have calcium availability at a level that would provide enough calcium to satisfy the needs of their populations ^32, 33^.

Calcium concentrations in water vary significantly according to the water source and geographic areas. For instance, calcium concentration of tap water varies from 1 to 135 mg/L across the US ^16^. The average calcium concentration of tap water is 50.6 ± 29.4 mg/L that is similar to most bottled spring waters ^16^. On the other hand, bottled mineral waters have much higher calcium concentrations, an average of 208 mg/L and could reach 500 mg/L ^16^.

In a previous study, using a national representative dietary intake data from Argentina (Second Health and Nutrition National Survey, ENNyS 2), we reported a high prevalence of low calcium intake, that reaches 88% of adult population ^13^. With this data we simulated increasing tap and bottled water concentrations and showed its contribution to recommended calcium intake ^33^. In a triangle test, we found that the sensory detection threshold of bottled water with added calcium salts allows the increase of calcium concentration of water up to a level of 500 mg of calcium/L ^34^. However, bottled water intake in Argentina seems to be low; thus, efforts to improve calcium content of other sources, such as tap water, is relevant. The calcium concentration of tap water in some cities of Argentina is low with an average calcium concentration between 12.9-19.0 mg/L depending on the region, and increasing this concentration could be explored to help an improvement in calcium intake ^32^.

There is a lack of knowledge about the maximum calcium level that could be added to tap water while complying with local regulations. Therefore, the objective of this study is to determine the maximum calcium that can be added to tap water complying with drinking water local regulations.

## Methods

This study was performed at a laboratory level using tap water directly obtained from the treatment water plant Aguas Bonaerenses S.A (ABSA) Donato Gerardi. This water plant has a capacity of 15,000 m3/h and supplies tap water to around 800,000 inhabitants of the Buenos Aires Province, Argentina. This conventional water plant uses surface water from Rio de La Plata that is treated with a combined process of coagulation, flocculation, sedimentation, filtration, and disinfection. It treats water in a central location and then distributes water via distribution networks.

### Physicochemical characterization of tap water

Drinking water quality in Argentina is regulated by the Food Codex of Argentina (Código Alimentario Argentino, CAA). This local regulation defines the maximum level of physicochemical parameters, inorganic, organic, biological, and radioactive substances for drinking water ^35^.

To characterise the tap water we collected from the treatment water plant a duplicate water sample of 1.5 L daily over 15 consecutive days. The purpose of this sampling strategy was to collect a representative sample following the general recommended collection of samples for water analysis by American Public Health Association (APHA) ^36^. Samples were taken from existing sampling locations at the final stage in the treatment plant where the variation in the parameters is expected to be minimal. Samples were obtained following standardised methodologies and physicochemical parameters of each water sample were assessed following analytical standardized methodologies ^36^.

Calcium concentration was measured by an atomic absorption spectrometer at 422.7 nm (Varian AA 240 FS) by direct air-acetylene flame method. Turbidity was obtained using a portable turbidimeter WGZ-2A (Shanghai Xinrui Instruments Co., Ltd). Colour was determined by visual comparison method and pH, water temperature (degrees Celsius, ºC), electrical conductivity, and total dissolved solids (TDS) were measured with Sper Scientific Water Quality Meter (Model 850081). Total water hardness was assessed according to the titrimetric method with ethylenediaminetetraacetic acid (EDTA). Chloride ion concentration was analysed by titration (Mohr’s Method) and total alkalinity, carbonates and bicarbonates were measured by titration method. TDS, pH, and conductivity were measured at the time of sampling while the other parameters were analysed within 24 hours after collection ^36^. Corrosive and scaling properties were estimated using the Langelier method. The saturation index provides precipitation or solubility tendency of calcium carbonate that defines if a water sample is over-saturated, saturated, or unsaturated ^37, 38^.

### Preparation of water sample with added calcium

To increase calcium concentration of water we used calcium chloride dihydrate that is a highly soluble inorganic salt (74.5 g per 100 ml at 20 °C) ^26^. We obtained the salt from Sigma-Aldrich (Cat#223506, Germany) that meets the analytical specifications of The European Pharmacopoeia (Ph. Eur.), Pharmaceutical Reference Standards (USP), FCC, E509.

We first theoretically estimated the amount of calcium that could be added to water using the results obtained in the water physicochemical characterization and the maximum total tap water hardness allowed by the CAA ^35^. With the estimated mean water hardness, we determined the theoretical amount of calcium chloride dihydrate to be added to the water samples to obtain solutions with hardness between 50 ppm up and 400 ppm of calcium carbonate, the maximum total water hardness allowed by the CAA ^35^.

We estimated that 4 L of sample water was required to prepare solutions with added calcium and perform duplicate analytic measurements. Of this volume, we took 3.5 L that was required. The 4 L of water were then divided into 14 aliquots of 250 ml each (C0, control duplicated sample) and the rest were used to add calcium chloride (C1 to C6, duplicated at each sample). Afterwards we determined all the physicochemical parameters and using the saturation index, we calculated again the corrosive scaling properties of each sample of water ^38^.

### Statistical analysis

The data analysis was carried out using SPSS statistical software package version 22.0 (SPSS Inc., USA).

## Results

The average physicochemical characterization of tap water samples obtained from the treatment water plant during 15 consecutive days is shown in Table 1. The mean calcium concentration was 22.00 ± 2.54 mg/L, the mean water hardness was 89.9 ± 6.4 mg/L CaCO3, and the saturation index was-1.50 ± 0.11 ( Table 1).

**Table 1.** Physicochemical parameters of baseline water from ABSA water treatment plant collected during 15 consecutive days.

Physicochemical parameters	Baseline water Mean (±SD) n= 15
Hardness (mg/L CaCO _3_)	89.9 ± 6.4
Calcium (mg/L Ca ^+2^) pH	22.00 ± 2.54 6.85 ± 0.11
Temperature (°C)	21.42 ± 1.69
Conductivity (mS/cm)	0.62 ± 0.05
Turbidity (NTU *)	< 1
Total alkalinity (mg/L CaCO _3_)	83.0 ± 16.50
Bicarbonates (mEq/L HCO _3 ^-^_)	1.63 ± 0.31
Carbonates (mEq/L CO _3 ^=^_)	< 0.08
Total dissolved solids (mg/L)	398.70± 40.90
Chloride ion (mg/L Cl-)	109.20 ± 10. 90
Colour (scale Pt-Co)	< 2.5
Langelier Index (LI)	-1.50 ± 0.11

*NTU: nephelometric turbidity units.

Table 2 shows the different physicochemical parameters of the sample water before and after adding calcium chloride dihydrate. The maximum calcium concentration obtained was 140.50 ± 2.12 mg/L when we added 0.4554±0.0071 g of salt. This water had a final water hardness of 355.0 ± 7.1 mg/L CaCO _3_ complying with CAA and a saturation index of -0.53 ± 0.02. We also obtained water with a calcium concentration of 67.30 ± 3.18 and a saturation index of -0.80 ± 0.01 when we added 0.1656 ± 0.0023 g of salt. This water had a hardness of 197.0 ± 1.4 mg/L CaCO _3_ ( Table 2) complying both with CAA.

**Table 2.** Physicochemical parameters of water samples before (C0) and after adding calcium chloride dihydrate in increased amounts (C1- C6).

	C0	C1	C2	C3	C4	C5	C6
Added calcium chloride (g CaCl _2_.2H _2_O/L water)	-	0.0980 ± 0.0028	0.1656 ± 0.0023	0.2358 ± 0.0042	0.3072 ± 0.0006	0.3834 ± 0.0008	0.4554 ± 0.0071
Calcium added(g/L)	-	0.0267 ± 0.0008	0.0451 ± 0.0006	0.0642 ± 0.0012	0.0837 ± 0.0002	0.1044 ± 0.0002	0.1241 ± 0.0019
Parameter							
Hardness (mg/L CaCO _3_)	90.0±1.4	151.0±1.4	197.0±1.4	236.5±5.0	280.0 ± 1.4	311.0± 1.4	355.0 ± 7.1
Calcium (mg/L Ca ^+2^)	22.00±1.41	48.50±0.71	67.30±3.18	91.00±2.83	108.00 ± 2.83	128.50 ± 2.12	140.50 ± 2.12
pH	6.990±0.014	7.095±0.007	7.125±0.035	7.140±0.014	7.090 ± 0.014	7.145 ± 0.007	7.115 ± 0.021
Temperature (°C)	22.60±0.14	22.85±0.07	22.90±0.14	22.85±0.07	22.85 ± 0.07	22.80 ± 0.14	22.75 ± 0.07
Conductivity (mS/cm)	0.615±0.021	0.765±0.007	0.860±0.004	0.965±0.007	1.060 ± 0.004	1.165 ± 0.007	1.265 ± 0.007
Turbidity (NTU)	<1.0	<1.0	<1.0	<1.0	<1.0	<1.0	<1.0
Total alkalinity (mg/L CaCO _3_)	73.0±0.7	73.0±0.7	74.0±1.41	73.0±0.7	74.0 ± 1.4	73.0 ± 0.7	75.0 ± 0.7
Bicarbonates (mEq/L HCO _3 ^-^_)	1.40±0.04	1.40±0.04	1.45±0.07	1.40±0.04	1.45 ± 0.07	1.40 ± 0.04	1.50 ± 0.04
Carbonates (mEq/L CO _3 ^=^_)	<0.08	<0.08	<0.08	<0.08	<0.08	<0.08	<0.08
Total dissolved solids (mg/L)	396.5±2.1	492.5±3.5	562.0±4.2	628.0±4.2	697.0 ± 5.7	767.5 ± 3.5	839.0 ± 1.4
Chloride ion (mg/L Cl-)	106.0±1.4	148.7±1.8	179.7±1.8	216.2±7.1	248.8 ± 3.4	292.8 ± 1.7	316.7 ± 7.1
Colour (scale Pt-Co)	<2.5	<2.5	<2.5	<2.5	<2.5	<2.5	<2.5
Langelier Index (LI)	-1.40±0.01	-0.97±0.01	-0.80±0.01	-0.70±0.01	-0.67 ± 0.01	-0.56 ± 0.01	-0.53 ± 0.02

Data are expressed in mean ± SD. NTU: nephelometric turbidity units

## Discussion

In our research we used tap water obtained from the treatment central water plant to determine the maximum amount of calcium that can be added. This study shows that, at laboratory level, it is feasible to increase the calcium concentration of a drinking tap water that originally had a calcium concentration of around 22 mg/L. This baseline level concentration was improved by adding calcium chloride; the calcium concentration of this tap water could reach 140 mg/L complying with the CAA which is the national tap water regulation in the country ^35^. We defined the maximum calcium level to be added as that level which complied with the local regulations of physicochemical parameters. This information would be required as a first step to start thinking in proposing the addition of a minimum standard of calcium concentration in tap water to local regulations. The current knowledge about calcium to be added to tap water comes from simulation studies and fortification of bottled water in other water sources ^39, 40^. Our research brings to light the maximun calcium that could be added to tap water while complying regulations in Argentina.

Water hardness and TDS increased with the addition of calcium chloride, but still complied with the tap water hardness and other physicochemical parameters regulated by the CAA ^35^. The CAA regulation allows a maximum total drinking water hardness of 400 mg/L, a maximum turbidity of 3 NTU, a maximum TDS of 1500 mg/L, a maximum chloride ion of 350 mg/L Cl, and maximum colour of 5 scale Pt-Co ^35^. On the other hand, the maximum level of chloride ion permitted by the Province of Buenos Aires is 250 mg/L, which is lower to the 350 mg/L permitted at national level. This regulation would limit the addition of calcium chloride, allowing increasing the calcium concentration of water from 22 to 100 mg/L ^41^. All physicochemical parameters measured before and after adding the salts indicate that the water complies with the water standards regulated by CAA ^35^.

Our results were obtained under controlled laboratory conditions to be able to perform all analytical tests and all water samples analysed were collected from the same point in the water treatment plant. The mean baseline water hardness was almost 90 mg/L CaCO _3_ indicating that the water is soft and had a general corrosive tendency with a saturation index of -1.5 ( Table 1). Corrosive tendency depends on physical and chemical characteristics of the water such as pH, total alkalinity, and hardness. The Langelier method is the widest method to determine whether water tends to precipitate CaCO _3_. Hence, this saturation index is not related directly to corrosion, but to the deposition of a calcium carbonate film or scale ^42^. After adding calcium chloride, the Langelier Index was maintained and the water samples were under-saturated.

The baseline water calcium concentration we report in this study is similar to the concentrations we previously reported from different areas of Argentina ^20^. Drinking water from water distribution plants tend to have low calcium concentrations as national regulations usually focus on the maximum hardness level to avoid scaling, but not on the minimum hardness level to avoid corrosion ^35^. In this way, water that complies with regulatory standards can have very low calcium concentration and be corrosive. Although previous epidemiologic studies have suggested an inverse relationship between water hardness and cardiovascular mortality, mainly determined by concentrations of calcium and magnesium, often drinking water guidelines do not base recommended hardness or TDS on health outcomes ^43– 48^.

According to World Health Organization (WHO) guidelines, calcium ion threshold taste is around 100–300 mg/L, depending on the associated anion; however, consumers could tolerate water hardness more than 500 mg/L ^42^. A study using water samples with similar characteristics to the sample used in this study showed that the sensory detection threshold of water with added calcium chloride dihydrate allowed an increase of calcium concentration of water up to a level of 291 ± 73 mg/L ^34^. TDS also contributes to palatability of drinking-water, and at TDS levels greater than about 1000 mg/L the water becomes significantly and increasingly unpalatable ^42^.

Even though water guidelines usually do not specifically refer to calcium concentration values, drinking-water can be a contributor to calcium intake and could be important for those who are marginal for calcium ^42^. In LMICs, including Argentina, the simulated strategy of increasing water with 500 mg of calcium/L showed that the prevalence of low calcium intake in all age groups could decrease without exceeding the recommended upper levels of calcium intake ^13^. Also, we have shown that the intake of one litre of drinking water from Argentina could represent on average between 1.2 and 8.0% of the calcium daily values for an adult ^20^.

In this study we propose that 140 mg of calcium per liter could be a maximum level; however in a previous study, we simulated adding 100 mg of calcium per liter of tap water considering real water consumption reported in the ENNyS 2. Using this data, we showed that the addition of 100 mg of calcium per liter of tap water represented a reduction of 11.3% percentage points in the prevalence of low calcium intake (from 91.0% to 79.7%) in women aged 19 to <31 years old and a reduction of 12.9% percentage points (from 89.4% to 76.5%) in women aged 31 to <51 years of age. In men, represented a reduction of 13.3% percentage points in the prevalence of low calcium intake (from 80.6% to 67.3%) in men aged 19 to <31, and a reduction of 15.6% percentage points (from 88.4% to 72.8%) in those aged 19 to <51 years ^33^. The reductions found in our simulations could be slightly increased taking into consideration the maximum level of 140 mg of calcium per liter that we assessed in this study ^33^. 

In this study, we analyzed calcium content of drinking water obtained from a centralized water treatment plant. Although our study was performed with water samples from only one treatment water plant, this plant provides tap water to the second most important agglomerate of Argentina. Our samples were taken at the final stage, before dedicated distribution networks. It is known that water characteristics could vary throughout the supply system. Thus, future research into the feasibility of water with added calcium should consider these potential changes. The inclusion of water quality analysis into distribution systems could apply developed sensor systems ^49^. A second approach could be to analyze the optimum stage of the water treatment process to add calcium. For instance, this procedure could include a detailed analysis of the water treatment process besides storage temperature, seasonality changes and pressure. Also, it could analyse characteristics of pipeline materials in the treatment and distribution systems. Furthermore, research into the feasibility of water with added calcium deserves to be considered since different water supplies and different baseline waters. The chemical composition of drinking water, including calcium concentration, is varied depending on the origin, treatment received, and distribution system ^50^.

In addition, we suggest that a possible approach to be implemented as large-scale addition of calcium chloride could be a point-of-use (POU) mineralization unit under the kitchen sink with a separate tap or a separate high calcium water line to the kitchen sink. However, adding calcium chloride after the POU installations or to naturally soft waters in a home presents some technical difficulties that must be resolved. Thus, this approach should be part of an implementation study. 

The drinking water quality data is limited in LMICs ^51– 53^. Drinking-water quality refers to physicochemical, organoleptic (taste-related), and biological characteristics of water based on standards ^42^. Water quality is one of four distinct types of health-based targets defined by WHO to protect human health ^42^. Drinking water services coverage has improved in all regions, however inequalities varied widely between LMICs ^52^. In Argentina, almost 90% of 31 urban agglomerates, including the area of our study, have accessibility to safe drinking water services ^54^, suggesting universal access is still not achieved. Moreover, in almost all LMICs the coverage gap between urban and rural areas can be seen. For instance, in Latin America significant disparities were estimated in coverage of safely managed drinking water between urban (81%) and rural (53 %) areas ^52^. National averages often mask significant inequalities in service levels within countries ^42, 55^. It would be valuable to count with disaggregated information about water composition and types of water supply systems in regions with low calcium intake to improve water quality characteristics to enhance calcium availability.

Calcium concentration of tap water could be increased if water is boiled. This higher concentration would not be affected by tap water regulations if boiling occurs after leaving the pipe system. Boiling water for at least 20 minutes effectively kills or inactivates most protozoa, bacteria, and viruses and minerals such as calcium get more concentrated as the water evaporates ^42, 56, 57^.

Considering the potential negative effects of drinking water with low calcium levels on the cardiovascular system setting a minimum calcium concentration of tap water should be contemplated.

## Conclusions

Calcium and water are essential elements for life, and their adequate intake is, therefore, vital for the maintenance of the body’s homeostasis. Calcium deficiency is high in many LMICs and multiple strategies should be developed in conjunction, to aid these populations attain an adequate calcium intake Also, adequate dietary calcium intake is necessary for the maintenance of bone health, and calcium-rich drinking tap waters in accordance with national standards can represent a valid strategy to reach this purpose.

The current research results could be a valuable and feasible resource to cast light on the discussion on standards for minimum calcium concentration in drinking- tap water. The research helps our scientific community to know about the maximum calcium level that could be added to tap water while complying with local regulations. Considering that calcium in water has a good bioavailability and that water is universally consumed, water with added calcium could help improve calcium intake in our country. Further assessments of the amount of calcium that could be added could be performed in each area of the country where it is demonstrated that calcium intake and calcium concentration of water are low as this could help improve calcium intake and prevent health risks.

## Data Availability

The data presented in this study are available on request from the corresponding author.
