# Supplementary material for: Feasibility of increasing calcium content of drinking tap water following quality regulations to improve calcium intake at population level
Source: Gates Open Res. 2024 Aug 29;8:5. Originally published 2024 Jan 30. [Version 2] doi: 10.12688/gatesopenres.15184.2 (PMC11421485; doi:10.12688/gatesopenres.15184.2)
Supplement: Peer review history

# Reviewer report: Reviewer response for version 2

**DOI:** 10.21956/gatesopenres.17683.r37835

**Published:** 2024-09-13

**Authors:** Giovanni Barone

**Affiliations:** 1 University of Copenhagen, Copenhagen, Denmark

The changes and responses to reviewers seem reasonable.

Is the work clearly and accurately presented and does it cite the current literature?

Yes

If applicable, is the statistical analysis and its interpretation appropriate?

Partly

Are all the source data underlying the results available to ensure full reproducibility?

Yes

Is the study design appropriate and is the work technically sound?

Yes

Are the conclusions drawn adequately supported by the results?

Yes

Are sufficient details of methods and analysis provided to allow replication by others?

Partly

Reviewer Expertise:

Food fortification, mineral ingredients, calcium fortification,

I confirm that I have read this submission and believe that I have an appropriate level of expertise to confirm that it is of an acceptable scientific standard.

# Reviewer report: Reviewer response for version 1

**DOI:** 10.21956/gatesopenres.16527.r36156

**Published:** 2024-05-14

**Authors:** Giovanni Barone

**Affiliations:** 1 University of Copenhagen, Copenhagen, Denmark

The work is fundamentally correct and well defined. However, most of the knowledge is already well established, and work doesn't bring any new helpful information. Moreover, there is a lack on how fortication can be implemented at large scale with sustainable source of calcium chloride. Besides, it would be interesting to investigate the available calcium after heat-treatment; as heat processing of water is mandatory in area where water are not microbiologically pure. 

 Background:

 A full stop is missing between ‘low The’

 Local regulation of where? It is very broad and too generic

 INTRODUCTION:

 Calcium accounts for 1 to 2 percent of adult human body weight. Over 99 percent of total body calcium is found in teeth and bones. Say that is most abundant elements makes no sense. Rephrase it.

 METHODS:

 4L dived in 14 aliquots makes 3.5L, can you clearly specify where is the rest? It is unclear.

Is the work clearly and accurately presented and does it cite the current literature?

Yes

If applicable, is the statistical analysis and its interpretation appropriate?

Partly

Are all the source data underlying the results available to ensure full reproducibility?

Yes

Is the study design appropriate and is the work technically sound?

Yes

Are the conclusions drawn adequately supported by the results?

Yes

Are sufficient details of methods and analysis provided to allow replication by others?

Partly

Reviewer Expertise:

Food fortification, mineral ingredients, calcium fortification,

I confirm that I have read this submission and believe that I have an appropriate level of expertise to confirm that it is of an acceptable scientific standard, however I have significant reservations, as outlined above.

# Response

**Published:** 2024-08-27

**Authors:** Maria B Puchulu

**Affiliations:** Facultad de Medicina, Departamento de Ciencias Fisiológicas, Universidad de Buenos Aires, Buenos Aires, Autonomous City of Buenos Aires, Argentina

*The work is fundamentally correct and well defined. However, most of the knowledge is already well established, and work doesn't bring any new helpful information.*

 Response: Thanks for your kindly comments and suggestions.

 We have added the following paragraph:

Discussion: In our research we used tap water obtained from the treatment central water plant to determine the maximum amount of calcium that can be added. This study shows that, at laboratory level, it is feasible to increase the calcium concentration of a drinking tap water that originally had a calcium concentration of around 22 mg/L. This baseline level concentration was improved by adding calcium chloride; the calcium concentration of this tap water could reach 140 mg/L complying with the CAA which is the national tap water regulation in the country ^35^. We defined the maximum calcium level to be added as that level which complied with the local regulations of physicochemical parameters. This information would be required as a first step to start thinking in proposing the addition of a minimum standard of calcium concentration in tap water to local regulations. The current knowledge about calcium to be added to tap water comes from simulation studies and fortification of bottled water in other water sources ^39,40^.  However, little is known about calcium added to tap drinking water. Thus, this research brings to light the real calcium added to tap water complying Argentine regulations.

*We have modified the conclusion section:*

Conclusions: Calcium and water are essential elements for life, and their adequate intake is, therefore, vital for the maintenance of the body’s homeostasis. Calcium deficiency is high in many LMICs and multiple strategies should be developed in conjunction, to aid these populations attain an adequate calcium intake. Also, adequate dietary calcium intake is necessary for the maintenance of bone health, and calcium-rich drinking tap waters in accordance with national standards can represent a valid strategy to reach this purpose.

 The current research results could be a valuable and feasible resource to cast light on the discussion on standards for minimum calcium concentration in drinking- tap water. The research helps our scientific community to know about the maximum calcium level that could be added to tap water while complying with local regulations. Considering that calcium in water has a good bioavailability and that water is universally consumed, water with added calcium could help improve calcium intake in our country. Further assessments of the amount of calcium that could be added could be performed in each area of the country where it is demonstrated that calcium intake and calcium concentration of water are low as this could help improve calcium intake and prevent health risks.

*Moreover, there is a lack on how fortification can be implemented at large scale with sustainable source of calcium chloride.*

 Response:

 Our research shows that at the laboratory level, it is feasible to increase calcium concentration in drinking water while complying with national standards, as the first step to proposing a large-scale study, adding calcium chloride.

*We discuss about this topic in the following paragraph:*

*Thus, future research into the feasibility of water with added calcium should consider these potential changes. The inclusion of water quality analysis into distribution systems could apply developed sensor systems* *^49^* *. A second approach could be to analyze the optimum stage of the water treatment process to add calcium. For instance, this procedure could include a detailed analysis of the water treatment process besides storage temperature, seasonality changes and pressure. Also, it could analyse characteristics of pipeline materials in the treatment and distribution systems. Furthermore, research into the feasibility of water with added calcium deserves to be considered since different water supplies and different baseline waters.*

 We have now added more options that require research.

*We have added the following paragraph to the discussion section:*

Discussion: In addition, we suggest that a possible approach to be implemented as large-scale addition of calcium chloride could be a point-of-use (POU) mineralization unit under the kitchen sink with a separate tap or a separate high calcium water line to the kitchen sink. However, adding calcium chloride after the POU installations or to naturally soft waters in a home presents some technical difficulties that must be resolved. Thus, this approach should be part of an implementation study. 

*Besides, it would be interesting to investigate the available calcium after heat-treatment; as heat processing of water is mandatory in area where water are not microbiologically pure.*

 Response:

 For this study we used water from one treatment plant that supplies tap water to around 800,000 inhabitants of the Buenos Aires Province.  Argentina is highly urbanized; 28 water treatment plants provide tap water to other districts of the Buenos Aires Province and three surface water treatment plants supply drinking tap water to more than 11 million inhabitants in Greater Buenos Aires, mostly from surface water. Thus, most homes in Buenos Aires have tap drinking water.

*We have added the following paragraph in the discussion section in red color:*

 Calcium concentration of tap water could be increased if water is boiled. This higher concentration would not be affected by tap water regulations if boiling occurs after leaving the pipe system. Boiling water for at least 20 minutes effectively kills or inactivates most protozoa, bacteria, and viruses and minerals such as calcium get more concentrated as the water evaporates ^42,^ ^56,57^.

 Background:

 A full stop is missing between ‘low The’

 Response: we have added a full stop (red color).

 Local regulation of where? It is very broad and too generic

 Response: we have added the word Argentine in red color; the final version of the background is:

 Calcium intake is below recommendations in several parts of the world. Improving calcium intake has benefits not only for bone health but also helps to prevent pregnancy hypertension disorders.  Calcium concentration of tap water is usually low. The aim of the present study was to determine the maximum amount of calcium that can be added to tap water while complying with drinking water Argentine regulations.

 INTRODUCTION:

 Calcium accounts for 1 to 2 percent of adult human body weight. Over 99 percent of total body calcium is found in teeth and bones. Say that is most abundant elements makes no sense. Rephrase it.

 Response: We have changed the sentence as follows:

*Calcium* *is one of the inorganic* *elements in the human body involved in many vital functions, influencing many extracellular and intracellular processes.*

 METHODS:

 4L dived in 14 aliquots makes 3.5L, can you clearly specify where is the rest? It is unclear.

 Response:

 It is known that during laboratory experiments, it is well established that the sample volume collected should be higher than the calculated volume. 

 In particular, to prepare solutions with added calcium and perform duplicate analytical measurements, we estimated that 3.5 liters of water were theoretically needed. We planned to take the water sample with a plus volume of 0.5 L to ensure the volume required for preparing the solutions in the laboratory. In this way, we obtained a 4 L of volume sample.

 We changed the paragraph as follows:

Methods: *We estimated that 4 L of sample water was required to prepare solutions with added calcium and perform duplicate analytic measurements.* *Of this volume, we took 3.5 L* *of water* *that* *was* *then divided into 14 aliquots of 250 ml each (C0, control duplicated sample) and the rest were used to add calcium chloride (C1 to C6, duplicated at each sample). Afterwards* *,* *we determined all the physicochemical parameters and using the saturation index, we calculated again the corrosive scaling properties of each sample of water ^38^.*

*We have modified the reference section.*

# Reviewer report: Reviewer response for version 1

**DOI:** 10.21956/gatesopenres.16527.r36153

**Published:** 2024-05-14

**Authors:** Isidro Vitoria

**Affiliations:** 1 Hospital Universitari i Politècnic la Fe, Valencia, Spain

The calcium in water has a bioavailability similar to the calcium in dairy products. The calcium intake of the population often does not reach the nutritional recommendations, especially at the present time when there is a lower consumption of dairy products. I think this article is very opportune in proposing calcium in drinking water as an adequate supplementary source of calcium. The study proposes to enrich the calcium content of drinking water in Buenos Aires, with a low hardness.The authors show that they can reach 140 mg/l of calcium and that these figures do not pose technical problems of taste or precipitation and saturation.The limitation of added calcium in relation to the water distribution system is also well stated.

 I think it would gain a lot if the authors would add a table indicating the percentage of the recommended calcium intake by age that could be achieved with waters of 100 and 150 mg/l of calcium.

Is the work clearly and accurately presented and does it cite the current literature?

Yes

If applicable, is the statistical analysis and its interpretation appropriate?

Yes

Are all the source data underlying the results available to ensure full reproducibility?

Yes

Is the study design appropriate and is the work technically sound?

Yes

Are the conclusions drawn adequately supported by the results?

Yes

Are sufficient details of methods and analysis provided to allow replication by others?

Yes

Reviewer Expertise:

Inborn errors of metabolism

I confirm that I have read this submission and believe that I have an appropriate level of expertise to confirm that it is of an acceptable scientific standard.

# Response

**Published:** 2024-08-27

**Authors:** Maria B Puchulu

**Affiliations:** Facultad de Medicina, Departamento de Ciencias Fisiológicas, Universidad de Buenos Aires, Buenos Aires, Autonomous City of Buenos Aires, Argentina

Response: Thanks for your kindly comments and suggestion. In the introduction section we report a high prevalence of low calcium intake in Argentina, and we agree with the reviewer on the importance of indicating the contribution to the percentage of the recommended calcium intake.

 As suggested by the reviewer, we have now added in the discussion section, more details from our simulation study where we report the potential contribution of adding calcium in tap water to calcium intake recommendations (reference number 33):  

**Discussion:** In this study we propose that 140 mg of calcium per liter could be a maximum level; however in a previous study, we simulated adding 100 mg of calcium per liter of tap water considering real water consumption reported in the ENNyS 2. Using this data, we showed that the addition of 100 mg of calcium per liter of tap water represented a reduction of 11.3% percentage points in the prevalence of low calcium intake (from 91.0% to 79.7%) in women aged 19 to <31 years old and a reduction of 12.9% percentage points (from 89.4% to 76.5%) in women aged 31 to <51 years of age. In men, represented a reduction of 13.3% percentage points in the prevalence of low calcium intake (from 80.6% to 67.3%) in men aged 19 to <31, and a reduction of 15.6% percentage points (from 88.4% to 72.8%) in those aged 19 to <51 years ^33^. The reductions found in our simulations could be slightly increased taking into consideration the maximum level of 140 mg of calcium per liter that we assessed in this study ^33^.

*We have also made some changes to the introduction section, so as not to repeat information we have now added with more details in the discussion. The final version is in red; in blue color and struck through is the text we have deleted.*
